# The Effects of a Dairy Probiotic Product, Espar, on Salivary Calcium and Mutans Streptococci

**DOI:** 10.5681/joddd.2013.023

**Published:** 2013-08-30

**Authors:** Hamidreza Poureslami, Lida Pishbin, Zahra Eslaminejad, Fatemeh Jahani Moqadam, Moshtagh Rashid Farokhi

**Affiliations:** ^1^Associate Professor, Department of Pediatric Dentistry, Faculty of Dentistry, Kerman University of Medical Sciences, Kerman, Iran; ^2^Assistant Professor, Department of Pediatric Dentistry, Faculty of Dentistry, Kerman University of Medical Sciences, Kerman, Iran; ^3^Associate Professor, Department of Microbiology, Faculty of Medicine, Kerman University of Medical Sciences, Kerman, Iran; ^4^Clinical Assistant Professor, Department of Comprehensive Dentistry, University of Texas, Health Science Center, San Antonio, USA

**Keywords:** Calcium, Streptococcus mutans, Probiotic, Saliva

## Abstract

***Background and aims.*** Espar is a dairy product of probiotic nature that contains useful bacteria and high calcium content. The aim of this study was to analyze effects of daily consumption of Espar on the number of salivary mutans streptococci and the level of calcium content in a population of 15 to 17 year-old female students.

***Materials and methods.*** A double-blind randomized crossover study (n = 50) of healthy female adolescents was implemented in four stage intervals. The first and third stages were ‘run-in’ and ‘wash-out’ intervals. For the second and fourth stages, two weeks long in duration, the participants consumed 100 grams of Espar or 200 grams of plain yogurt. At the end of each stage, the number of salivary mutans streptococci and the level of calcium content were documented.

***Results.*** There was a statistically significant decrease in the number of salivary mutans streptococci subsequent to Espar consumption when compared to ordinary yogurt (p < 0.01). Additionally, salivary calcium content increased significantly subsequent to the consumption of Espar and yogurt. However, Espar yielded a higher level of significant increase in salivary calcium when compared to plain yogurt (p < 0.01).

***Conclusion.*** This study found that daily consumption of Espar increased the salivary calcium level while decreasing mutans streptococci of the saliva.

## Introduction

Despite the widespread use of fluoride in efforts to reduce the prevalence and severity of dental caries, it remains in the domain of the most common infectious diseases.^1,2^ Currently with the advent of bacterio-therapy, probiotics offer a possibility of changing the level of pathogenic bacteria present in the oral cavity and dental plaque. Probiotic is a bacteria-containing food product that induces desirable effects on the host through balancing the microbial flora. Consumption of probiotics such as milk and yogurt is currently considered an appropriate therapeutic method in preventing dental caries.^3^

The calcium concentration of dental plaque and saliva is an important index accounting for the balance between stages of de-mineralized and re-mineralized enamel. Numerous studies have shown that consumption of milk and yogurt can reduce the risk of dental caries through high calcium concentrations.^4^

Espar, as a traditional probiotic dairy by-product of yogurt, contains high levels of calcium and is exclusive to Shahrbabak, Iran. Initially, yogurt is poured and kept in specially designed containers for up to five days. Later, addition of equal part of water to the yogurt in the container allows for severe agitation of the container for up to two hours. The remainder of the liquid is boiled to extract the fat contents, hence; the condensation process. Subsequently, the condensed liquid poured into a special fabric bag allows for the evaporation of water contents. Then an equal part of liquid yogurt replaces that of the lost water at a 4-day interval. In one month, the final by-product or Espar is ready for daily consumption as breakfast, lunch, dinner or simply a snack between meals. It is important to note that during this entire process, and in particular the fermentation stage, the useful bacteria content is increased for Espar.

Epidemiological reports from Shahrbabak Health Center, regarding the prevalence of caries in 6, 12, and 15 year-old children have documented a prevalence rate of 37 percent, 21 percent, and 45 percent respectively (News Bulletin of Shahrbabak Health Center, Summer 2007). While a similar study in Kerman, located 150 kilometers from Shahrbabak, indicates the prevalence of caries of approximately 80 percent, 73 percent, and 21 percent respectively for the same age groups.^5^ Given the fact that the fluoride content of drinking water is similar between these two cities with 0.3 ppm in Kerman and 0.4 ppm in Shahrbabak,^6^ fluoride has a meager effect on such differences amongst the caries experience rates. Furthermore, after accounting for the similar oral hygiene and life style behaviors of these two populations, the authors are hypothesizing that the consumption of higher amounts of dairy products especially Espar in Shahrbabak seems to be a determining factor regarding the lower caries percentage observed compared to that of Kerman. This study aims to evaluate the effect of Espar on the number of the salivary mutans streptococci as well as the levels of salivary calcium.

## Materials & Methods

The Ethics Committee at the Kerman University of Medical Sciences, approved this proposed study, Ethical code K/88-68, as a double blind randomized cross over study. The Sample size was the random selection of fifty female students aged 15 to 17 old years who volunteered to participate in the study. Informed consent was obtained in the format of oral and written protocol from all participants who were residents of a boarding school in Kerman. Additionally, the Kerman School District granted written permission and protocol regarding this study.

Inclusion criteria for the participants included:^7,8^ absence of any systemic diseases, absence of any antibiotic use for one month prior to the study protocol initiation, lack of any mouth rinse or fluoride gel use, and lack of any use of xylitol containing gums up to a month prior to the study start date. Furthermore, the volunteer participants should not have any cavitated dental lesions in addition to no alterations in their existing daily oral hygiene and tooth brushing habits in order to qualify.

**figure1:**
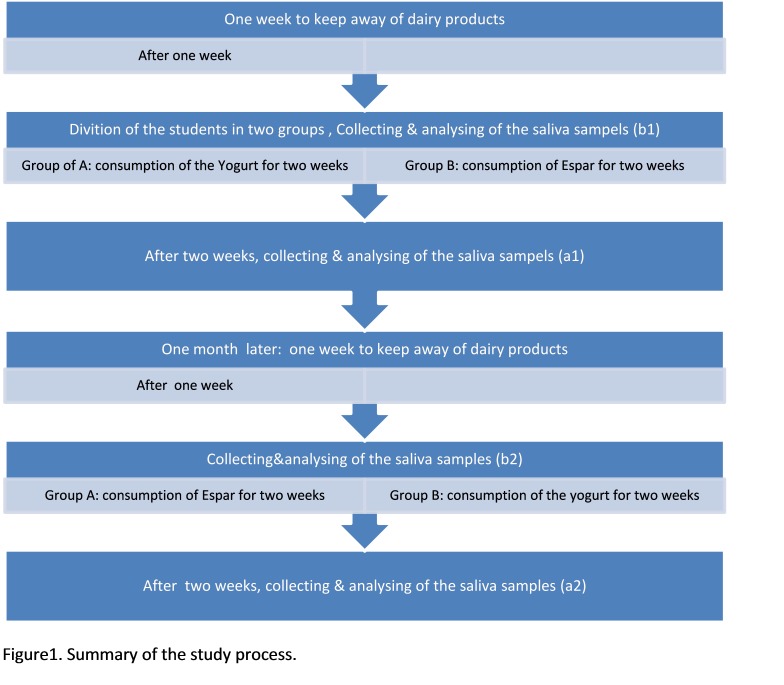
Summary of the study process.

Study process was comprised of four stages, b1, b2, a1, a2 ( Figure 1).^9,10^ At the first stage, b1 or ‘Run-ins’, the participants were prohibited to use any condensed dairy product such as yogurt, condensed yogurt or cheese altogether and were instructed to follow their daily oral hygiene regiment. At one-week interval, the participants were divided into two groups using random numbers, and were required to submit a one-minute salivary sample in a coded capped container at 6:30 AM, an hour prior to their breakfast.

The second stage or a1 included two sub-groups of participants selected randomly prior to the start of the experiment. With prior agreement, the boarding-school officials fed one group of participants 200 g of plain yogurt compared to the other group that consumed 100 g of Espar in addition to their regular menu for a two-week duration. Both products were produced by the same manufacturer (Shaban Factory, Sharbabak, Iran). The un-stimulated salivary samples were collected at the end of this stage.

During the third stage or the wash out stage, participants followed their regular daily diets for one month. After this period or b2, the participants were prohibited from consuming any condensed dairy products for a week and their non-stimulated saliva samples were collected.

At the fourth and final stage of a2, the groups switched the order of consumption where the yogurt group consumed 100 g of Espar and the Espar group consumed 200 g of plain yogurt for two weeks. Two weeks later non-stimulated saliva samples were collected again. Upon the conclusion of this study, an analysis of the entire collection of salivary samples during all four stages was completed.

### Measuring Saliva Calcium

The calcium content of saliva was measured using photometry method in the wavelength of 570 nanometers in RA1000 analyzer with the Pars Azmoon Co. quantity diagnosis kit. The scale used was Mill mol calcium in a Liter of saliva. The use of Cresolphtalein Complexone (CPC) allowed for a certain volume of homogenized saliva sample to move into an alkaline environment. Within such environment, calcium turns into a purple complex where the degree of color intensity depended upon the calcium contents of the samples. The colorful solution then underwent spectrophotometry where saliva calcium content was determined in units of Mmol/L using special kit.^11^ It was noted that the CPC technique can yield a slight overestimation to that of Atomic Absorption Spectrometer (AAS) because of affinity to Magnesium.

### Measuring the Number of Mutans Streptococci

Initially, the salivary samples homogenized by the way of mechanical shaking technique. Next, the addition of 25 micrograms of Neomycin to each container assured the inhibition of other-undesired-bacteria growth where the samples incubated at 37°C for 2 hours. A serial dilution was prepared from each sample and 20 µl of 3 with the final dilutions placed on Blood agar plates (Himedia, India) for cultivation. Later, the plates incubated in a Candle jar containing 10% CO_2_ at 37°C, for 48- 72 hrs.^12^ Identification of the mutans streptococci was by the use of standard methods such as colony character, Gram stain, Bacitracin and Optochin sensitivity, Mannitol, VP, and ONPG tests.^13^ Finally, the numbers of colonies were accounted for each plate where dilutions and/ or cultivations were repeated if indicated.

### Statistical Analysis

The data were analyzed using SPSS version 16. The reported salivary numbers of mutans streptococci was on a cardinal basis with the calcium content as milligrams in deciliters. The Student's t-test assessed the relationship of mutans streptococci of the saliva and calcium content following the consumption of each of the two distinct dairy products. Additionally, the effect of these dairy products on the number of mutans streptococci and calcium content of saliva were compared.

## Results

Throughout the duration of this project, three students changed their mind about collaboration therefore; the final number of participants was at 47 students. The Espar used for this study contained 25 × 10^8^ colonies of lactobacilli per one gram whereas the controlled plain yogurt product contained 4 × 10^6^ lactobacilli. The Espar contained 8 mg/g calcium whereas the study plain yogurt contained 0.9 mg/g.

There was no statistical significance between the average numbers of mutans streptococci before and after plain yogurt intake (P = 0.11; Tables 1 & 3). However, there was a significant difference in average salivary calcium level before and after plain yogurt intake (P = 0. 01; Table 1 & 4).

**Table 1:** Number of mutans streptococci (ms) and calcium content of saliva for the four stages of the study subsequent to consumption of plain yogurt

Test	Before intake, b1	Before intake, b2	After intake, a1	After intake, a2
Measuring MS (Colonies)	1.588×10^6^	1.630×10^6^	1.101×10^6^	1.125×10^6^
Measuring Calcium(mmol/L)	2.10	2.28	2.31	2.57

**Table 3:** Numbers of mutans streptococci (ms) in saliva subsequent to consumption of yogurt and Espar in colony forming units

Dairy product	Mean before intake (SD)	Mean after intake(SD)	P Value
Yogurt	1.609×10^6^(1.309×10^6^)	1.113 ×10^6^(1.023 ×10^6^)	0.11
Espar	1.899 ×10^6^ (1.021 ×10^6^)	0.407 ×10^6^ (0.203 ×10^6^)	0.00

**Table 4:** Table 4. Changes in Saliva Calcium Content Subsequent to Consumption of Yogurt and Espar in mmol/L

Dairy product	Mean before intake (SD)	Mean after intake (SD)	P Value
Yogurt	2.19 (0.85)	2.44 (0.67)	0.01
Espar	2.12 (0.62)	3.04 (0.75)	0.00

The difference between the average numbers of mutans streptococci before and after consumption of Espar was significant (P = 0.000; Tables 2 & 3). There was also statistical significant differences in average salivary calcium level before and after Espar intake (P = 0.000; Tables 2 & 4).

**Table 2:** Number of mutans streptococci (ms) and calcium content of saliva for the four stages of the study subsequent to consumption of Espar

Test	Before intake, b1	Before intake, b2	After intake, a1	After intake, a2
Measuring MS (colonies)	1.910×10^6^	1.888×10^6^	0.422×10^6^	0.392×10^6^
Measuring Calcium(mmol/L)	2.19	2.05	3.13	2.95

Comparing Espar intake to the plain yogurt, there was a decrease in the number of salivary mutans streptococci at a significance level (P < 0.01). Salivary Calcium content increased significantly subsequent to the consumption of both Espar and plain yogurt. However, Espar yielded a higher increase in salivary calcium levels when compared to plain yogurt (P < 0.01).

## Discussion

Bacteriotherapy or the use of harmless bacteria to displace pathogenic organisms is a promising way of combating infections. The present study is of probiotic nature. Probiotic, a Greek word meaning “towards life,” is a food product containing bacteria, which exerts desirable influences on the host by balancing his/her digestive microbial flora.^14^

This study attempted to promote how daily consumption of 100 g of Espar, for two-week duration can lead to a significant decrease in the average number of salivary mutans streptococci levels. In contrast, the daily consumption of 200 g of plain yogurt for two-week duration did not produce such significance. Since Espar studies are nonexistent, it is difficult to compare the current study results with that of others. In a cross over study by Caglar et al^15^ stated that consumption of probiotic yogurt was significant in decreasing the number of mutans streptococci whereas for plain yogurt consumption such decrease was not significant. Caglar et al^16^ also documented that consumption of probiotic ice-cream in comparison to non-probiotic ice-cream decreased salivary mutans streptococci significantly. Clidir et al^17^ conducted a similar study using two types of probiotic and plain yogurt where it concluded that the number of mutans streptococci only decreased significantly with consumption of the probiotic yogurt group. Comparison of the current study with the above studies indicates that Espar, as a justifiable probiotic product, has a number of useful bacteria when compared to that of the plain yogurt.

Within the design of this study, Espar and the plain yogurt intakes both showed a significant increase in the level of salivary calcium whereas such increase was much higher for the Espar intake. Comparison of current study with above studies indicates that Espar is a justifiable probiotic product with a great number of useful bacteria in comparison to the plain yogurt.

In the current study, while Espar and the plain yogurt intake both showed a significant increase in levels of salivary calcium, Espar intake led to much higher increase due to a higher calcium concentration. Consumption of high-calcium dairy products can be an effective method in decreasing dental enamel demineralization by increasing the calcium content of dental plaque. Higher levels of salivary calcium increase the re-mineralization potency of the plaque.^4,11^ Supportive studies, have documented that cheese consumption leads to an increase in calcium concentration of dental plaque, Moynihan,^4^ and daily consumption of cheese significantly increases the concentration of salivary calcium, Jenkins.^18^ Despite the difference of dairy products tested in the current study when compared to the above studies, present study supports similar findings of an increase of salivary calcium levels with the consumption of Espar.

Calcium content of un-stimulated whole saliva is reported as 0.5 to 2 mmol/L.^19,20^ Within the current study design, calcium content of un-stimulated whole saliva samples was around 2 mmol/L prior to the intake of the dairy products, where as it was raised to 2.44 and 3.04 mmol/L respectively after the intake of plain yogurt and Espar. Calcium favors enamel re-mineralization and reduces de-mineralization. Furthermore, calcium has a positive relationship with fluoride levels in microbial plaque and can diffuse into plaque and provide extra binding sites for fluoride.^20^ One study has shown that fluoride and calcium ions had positive effects on controlling of dental caries independent of each other.^20^ Calcium content of Espar is around 8mg/g and after Espar consumption salivary calcium contents rise up to 3.04mmol/L. Therefore, Espar is an excellent source of calcium delivery in the oral environment.

## Conclusion

Daily consumption of Espar decreases the number of cariogenic bacteria in the saliva compared to that of the plain yogurt. Thus, Espar can be a preventive product targeting dental caries in efforts towards enamel re-mineralization by increasing concentration level of salivary calcium content.
